# A dense-sampling investigation of interoceptive variation across the menstrual cycle: the Cyclic Hormones and Interoception with Repeated Profiling (CHIRP) Study

**DOI:** 10.1016/j.bbih.2026.101363

**Published:** 2026-09-16

**Authors:** Paul W. Savoca, Savannah D. Lopez, Sahib S. Khalsa, Bridget L. Callaghan

**Affiliations:** a Department of Psychology, University of California Los Angeles, Los Angeles, CA, 90095, United States of America; b Department of Psychiatry and Biobehavioral Sciences, UCLA Semel Institute for Neuroscience and Human Behavior, Geffen School of Medicine, Los Angeles, CA, 90095, United States of America

**Keywords:** Interoception, Dense sampling, Women's mental health, Hormones, Neuroimaging

## Abstract

The menstrual cycle is associated with variation in psychological and sensory processing and can exert substantial influence on mental health. One process which may be impacted by hormonal fluctuations during the menstrual cycle is interoception – the process by which the nervous system senses, integrates, and models the internal state of the body. However, limited work has examined if and how interoception may change across the menstrual cycle and if these changes are associated with mental health outcomes. Additionally, outstanding questions surrounding the reliability and concordance of interoception measures over time hinder progress in understanding these associations. To address these limitations, the CHIRP study aims to provide an illustrative example in two participants of how measures of interoception may vary across the course of the menstrual cycle using a dense sampling approach that spans multiple organ systems and dimensions of interoception (e.g., self-report and task-based measures), focusing on associations between fluctuations in interoception and mood. The repeated measures will be analyzed using Generative Additive Models (GAMs) to assess fluctuations across the cycle. Ultimately, this project aims to provide insight into how reproductive hormones may shape interoceptive processing and generate hypotheses for future investigations of menstrual-cycle–related mental health risk.

## 1 Introduction

Interoception is the process by which the nervous system senses, integrates, and models the internal state of the body (Craig, 2002; Khalsa et al., 2018; Sherrington, 1952). A growing body of evidence has begun to link individual variation in interoception to disturbances in mood such as depressive (Avery et al., 2014; Barrett et al., 2016; Brand et al., 2023; Eggart et al., 2019) and anxious symptoms and pathology (Domschke et al., 2010; Lapidus et al., 2020; Paulus, 2013; Quadt et al., 2021; Saltafossi et al., 2024; Verdonk et al., 2024). Additionally, there is existing evidence which highlights sex differences in both interoception (Prentice et al., 2022) and prevalence of mood disorders (Rubinow and Schmidt, 2019). The exact means by which interoception and mood are linked remained unclear, but several potential pathways have been hypothesized, including trait-level differences in awareness of interoceptive signals, state-level differences in physiological context and regulation (i.e., allostasis), exteroceptive context (i.e., visual, auditory, and olfactory cues), and alterations in predictive processing of interoceptive signals (Joshi et al., 2023). Additionally, the individual differences which influence these links have yet to be well characterized. This may, in part, be driven by the fact that many of the proposed influences on interoceptive individual differences, such as early-life adversity exposure (Bonaz et al., 2021; Ditzer et al., 2025; Schaan et al., 2019), traumatic experiences (Joshi et al., 2023), and hormones (Murphy et al., 2017; Savoca et al., 2024), are difficult to ethically manipulate. However, experimental manipulation often allows for the greatest scientific insight into underlying mechanisms. As such, we plan to use the fluctuations of hormones across the menstrual cycle as a natural experiment to probe if hormonally driven changes in bodily signaling are associated with altered interoceptive inference and if these changes follow alterations in mood.

### 1.1 The menstrual cycle and hormonal fluctuations

The menstrual cycle can be divided into several phases that are marked by significant fluctuations in several hormones including estrogens, progesterone, and luteinizing hormone (LH; Schmalenberger et al., 2021). The first, or ‘follicular’, phase begins on the first day of menstruation and continues until ovulation (Schmalenberger et al., 2021). The follicular phase is characterized by low levels of progesterone with increasing levels of estradiol (E2) in the later portion of this phase which peak just prior to ovulation. During the luteal phase there is a significant increase in progesterone (P4) levels that peak at the middle of the phase, and then decline in the second half of the phase (Schmalenberger et al., 2021). While there are minor fluctuations of E2 during the luteal phase, sensitivity to changes in P4 levels and its metabolites are thought to be critical for physiological and mood disturbances associated with the menstrual cycle (Wei et al., 2018). This later portion of the luteal phase, also referred to as the premenstrual phase, is when many menstruating women experience premenstrual symptoms (e.g., premenstrual syndrome; PMS). PMS symptoms, which include mood disturbances, cravings, and physical discomfort, are estimated to have a moderate or severe impact on the ability to perform activities of daily life (ADLs) in nearly 35% of menstruating women (Dennerstein et al., 2010). Also occurring during this same timeframe are symptoms of Premenstrual Dysphoric Disorder (PMDD; Wei et al., 2018). PMDD has a much more severe impact on mental health including emotion dysregulation, depression, and anxiety – affecting an estimated 3-7% of menstruating women globally. Our hypothesis is that fluctuations in hormones could be associated with variations in the ability to sense, perceive, and experience bodily sensations, which in turn could influence the severity of PMS/PMDD symptoms.

### 1.2 Pathways linking the menstrual cycle with interoception and mood

Below, we speculate on three separable, but not independent, pathways by which the menstrual cycle could influence interoception and mood: 1) phasic changes in central interoceptive predictive processes, 2) changes in afferent interoceptive signals, and 3) trait-level differences in interoceptive awareness.

#### 1.2.1 Phasic changes in central interoceptive predictive processes

We propose that hormone associated changes in central interoceptive predictive processes would depend on levels of progesterone via a key metabolite: allopregnanolone. Allopregnanolone is synthesized from progesterone (via 5α-reductase and 3α-hydroxysteroid-dehydrogenase) both centrally in the brain and peripherally in the adrenal glands and ovaries (Kimball et al., 2020). We suggest that allopregnanolone influences central interoceptive predictive processing via effects at the level of GABA neurons in the insula – a primary integration point for interoceptive predictions and prediction errors (Barrett and Simmons, 2015). Empirical research has shown greater levels of GABA in the insula to be associated with interoceptive processing and greater cardiac interoceptive accuracy (Wiebking et al., 2014). While allopregnanolone does not change concentrations of GABA, it does improve the efficacy of GABA neurons, which has been suggested to be involved in inhibitory signaling that improves the precision of interoceptive prediction errors (Savoca et al., 2024). We would therefore expect reductions in allopregnanolone (which decreases from the luteal to follicular phase; Kimball et al., 2020) to be associated with reduced interoceptive precision.

#### 1.2.2 Changes in afferent interoceptive signals

Changes in the afferent signals of the body across the menstrual cycle may also influence fluctuations in interoception and mood. For example, during late-luteal and into the early-follicular phase there is an increase in perceivable uterine contractions (Bulletti et al., 2004). In this case, greater awareness of interoceptive signals coming from uterine contractions due to greater interoceptive signal (presence, or increased power, of uterine contractions) would be possible. There is also evidence for changes in other bodily systems, such as the gastrointestinal system, which has been shown to differ in function and symptom report across the menstrual cycle (Judkins et al., 2020). Another possibility is that uterine contractions during the late luteal-early follicular phase may compete with interoceptive signals originating from other organ systems, causing relative shifts in interoceptive accuracy across different organ systems. Furthermore, these less frequent sensations of uterine contractions, paired with an allostatic perturbance (e.g., blood loss and hemostasis), may increase attention to the contractions or other relevant afferent signals (accurate perception and attention are separable aspects of interoception; Murphy et al., 2020) and may increase the salience of these signals. Finally, the ability to localize sensations in the periphery may be limited (Shaffer et al., 2023), so we may observe shared attention and awareness for relatively close points of origin (e.g., coupling between gastric and uterine sensations during late luteal and early follicular phases).

#### 1.2.3 Trait-level differences in interoceptive awareness

Finally, there may be trait-level differences in interoceptive awareness (primarily self-reported beliefs and insight) between individuals, that we would not expect to change across the menstrual cycle, but that might moderate how an individual experiences physiological changes across the menstrual cycle. Particularly, differences in the categorization or interpretation of bodily signals between individuals could be amplified when there are fluctuations in the underlying physiological cues. For example, self-reported trait differences in ‘not-worrying’ and ‘trusting’ of bodily sensations have been associated with lower PMS symptoms, while ‘noticing’ and ‘emotional awareness’ of bodily sensations were associated with greater PMS symptoms (Borlimi et al., 2023). In this case, women who trust and do not worry about their body signals may experience less distress from the physiological changes associated with the pre-menstrual phase, while those who are more likely to notice these changes or use body sensations to inform their affective state could be more susceptible to reporting or experiencing symptoms and changes in mood. If this were true, we would expect to see stable differences in interoceptive awareness across the menstrual cycle between individuals who experience higher and lower premenstrual mood symptoms.

### 1.3 Significance of dense sampling

While research studies often strive to increase sample sizes in an effort to gain statistical power and increase the generalizability of findings, high-density sampling within small samples, so called ‘dense sampling’ (Poldrack et al., 2015) is gaining traction as an alternative method to address power, and has distinct advantages over ‘larger-sparser’ studies. Similar to a traditional longitudinal study design, dense sampling designs take repeated samples of the same participants, but diverge with respect to how many samples are taken and over what timeframe – dense designs usually take many more samples over much shorter timeframes than traditional longitudinal designs (Pritschet et al., 2021). This dense sampling approach can better capture temporal ordering and dynamic within person processes. As such, studies of psychological, hormonal, and neurological variation across the menstrual cycle may particularly benefit from dense sampling approaches (Heller et al., 2025). Indeed, dense sampling of neuroimaging data across the menstrual cycle has revealed subtle but meaningful variations in brain networks which appear to be related to cyclical fluctuations in hormones (Pritschet et al., 2020). While some of these neural changes have been reported to occur in regions that overlap with those involved in interoception (e.g., default mode network regions), to date, no study has applied a dense sampling approach to study variations in interoception across the menstrual cycle.

### 1.4 The cyclic hormones and interoception with repeated profiling (CHIRP) study

To further examine the pathways via which the menstrual cycle influences interoception and mood, we designed the Cyclic Hormones and Interoception with Repeated Profiling (CHIRP) study, which aims to address the knowledge gap of if and how interoception varies across the menstrual cycle within an individual, and if there are differences in the coupling of interoception and affect associated with PMDD. To improve modeling of these dynamic within-person processes, the study employs a dense sampling approach. To establish specificity of within-person interoception change we included measures of interoception that span different dimensions (e.g., sensibility, accuracy, and insight) and organ systems (e.g., cardiovascular, respiratory, and gastrointestinal, uterine). To assess links to mood, and gauge clinical utility, we will follow two naturally cycling female participants, one of whom was recruited on the basis of having elevated PMDD symptoms and the other with no elevations in these symptoms. Over the course of 4 months, these females will track their ovulation to determine stage of the cycle and will be assessed approximately every other day (52 in-person sessions total) on measures of interoception and mood, as well as undergo several neuroimaging sessions.

Interoception will be assessed via self-report and task-based measures that span several organ systems. This includes tasked-based assessments of cardiac, respiratory, and gastrointestinal interoception, and also neural measures of interoceptive attention – including attention to uterine sensations – using functional Magnetic Resonance Imaging (fMRI). Additionally, we will collect task-based assessments of exteroceptive sensory processing including taste, touch, and visual processing. Along with self-report measures of interoception and mood, and task based-measures of interoception, the study also will collect a variety of biological measures including structural MRI, gut-microbiome samples, resting heart rate, and LH levels.

Aim 1: Assess whether interoceptive processing exhibits phase-specific modulation across the menstrual cycle. Across interoception measures, we hypothesize that there will be significant differences in interoception between phases of the menstrual cycle, with the late luteal phase being most associated with poor task performance and high worrying. An exploratory arm of this aim is to determine correlations between interoceptive measures across organs, and to determine if variation in interoception within a particular organ system/s is more or less tied to the menstrual cycle phase than others.

Aim 2: Characterize whether menstrual-cycle–related shifts in interoception are associated with concurrent mood and subsequent mood fluctuations. We hypothesize that phase-related reductions in task-based assessments of interoceptive accuracy will be associated with worse reported mood (i.e., negative affect, depressive and anxious symptoms), while increases in the worrying about bodily sensations will also be associated with worse mood symptoms. We will also test if these associations are specific to a given organ system.

Aim 3: Investigate whether PMDD is associated with heightened sensitivity of interoceptive systems to hormonal fluctuation within an individual. We hypothesize during the late-luteal phase, the PMDD participant will show a greater mismatch between task-based performance and self-reported interoception (relative to other phases) and greater coupling between self-report interoception and negative affect (relative to the control participant).

Exploratory Aims: Given the existing evidence for gastrointestinal changes across the menstrual cycle, this study also plans to characterize gut-microbiome dynamics in relation to interoception across the cycle. We plan to test, for the first time using a dense sampling approach, if the composition of the gut-microbiome varies across the menstrual cycle, and links between the microbiome and interoception across the menstrual cycle. As this is a completely novel aim, we do not have specific hypotheses and will engage only in exploratory analyses.

## 2 Methods

### 2.1 Pre-study procedures

The study employs a dense sampling approach to understand variations in interoception across the menstrual cycle. Prior to the first month of data collection, participants will complete daily home urine assessments of luteinizing hormone (LH) to anchor the stage of their menstrual cycle using *Premom* testing kits and the associated mobile phone app. Reminders to complete and upload the results of their LH testing will be sent daily at 6am. Participants will be encouraged to complete the testing at the same time each day. Participants will continue to complete LH testing at home throughout the study, but testing will be limited to the end of menstruation through several days after their LH peak (i.e., ovulation). Researchers will be able to check the completion of LH testing daily, in order to follow-up on missed samples. Prior to in-person data collection participants will also complete a one-time baseline assessment over Zoom. During this session they will complete the Mini International Neuropsychiatric Interview (M.I.N.I.; Sheehan et al., 1998) and a set of questionnaires on REDCap (Research Electronic Data Capture; hosted at UCLA; CTSI Grant UL1TR001881; Harris et al., 2019) which assess a variety of domains such as depression, anxiety, self-report interoception, and early-life adversity.

### 2.2 Study procedures

**Fig. 1:**
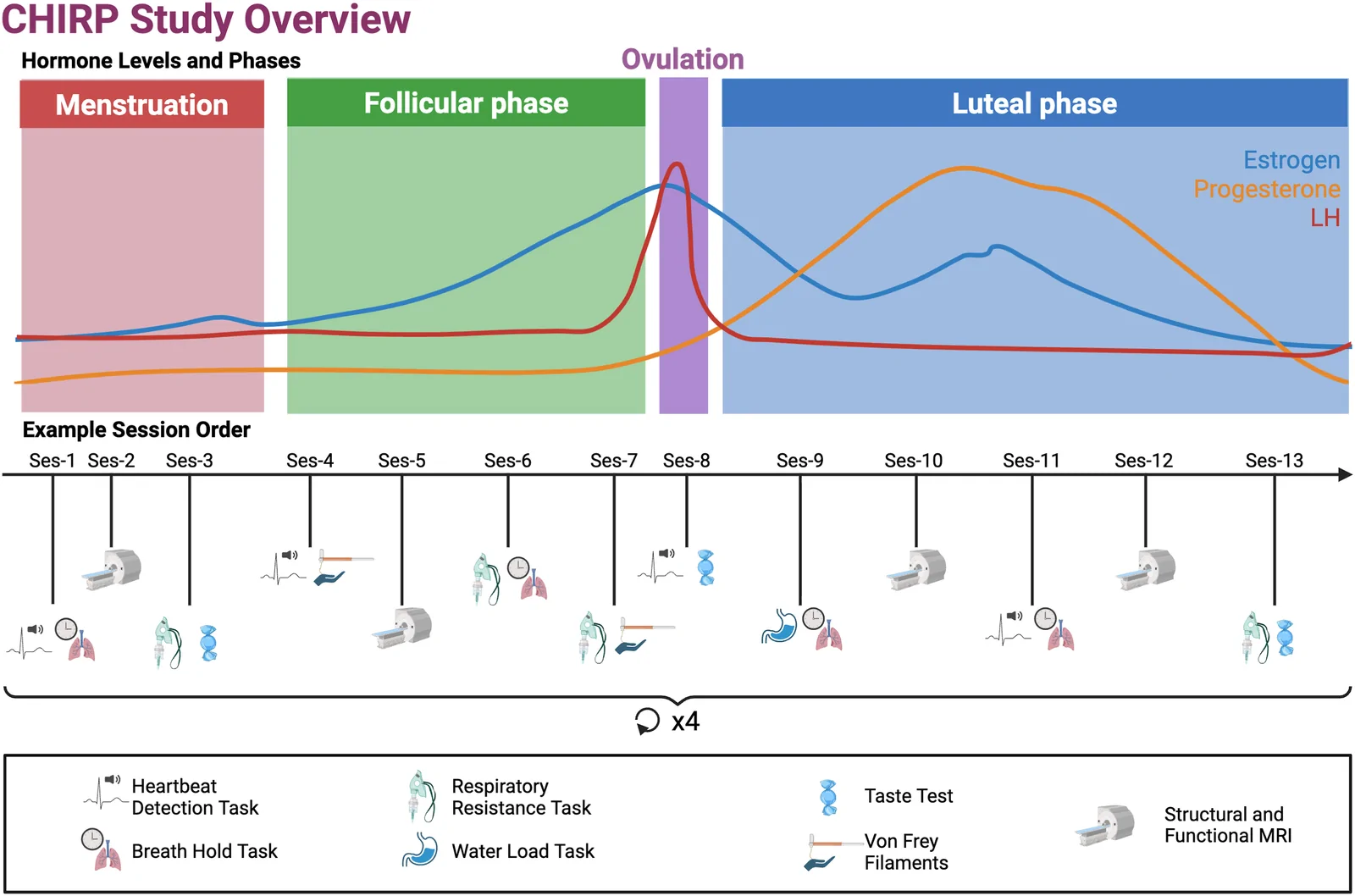
Study Overview. An example of order of sessions is depicted above. Participants completed sessions approximately every other day over the course of a month. Across months, session types were balanced to attempt to capture all phases of the menstrual cycle. Abbreviations: Magnetic Resonance Imaging (MRI).

For the in-person data collection, participants will complete sessions approximately every other day for 4 months (13 in-person sessions per month; see Fig. 1). The 4 months will not need to be completed consecutively, but all months need to be completed within 1 year of enrollment. Each in-person session will be either a Lab session (9 per month) or an MRI session (4 per month). In total, the study includes 52 in-person sessions (36 Lab sessions; 16 MRI sessions). All sessions will be scheduled approximately at the same time of day for each participant, and the same research staff will be present at all sessions. To control for the effect of researcher, the same researcher (PWS) will conduct all the research sessions. For MRI sessions, a secondary researcher is needed for safety procedures and again will be the same for all sessions (SDL). All research procedures are approved by the University of California, Los Angeles (UCLA) Institutional Review Board (IRB; #24-6193).

### 2.3 Lab sessions

During the lab sessions, the participants will first complete the state-based questionnaire on an iPad. The researcher will then share with the participant which two tasks they will complete during that session. Participants will then complete one of the three primary interoceptive tasks (Heartbeat Detection, Water Load, or Respiratory Resistance) followed by one of the three secondary tasks (Breath Hold, Taste Test or Von Frey Filaments). All tasks will be assigned in a semi-random fashion across days, ensuring that the tasks are performed a similar number of times across different phases of the menstrual cycle. All questionnaires and lab tasks are described in detail below (in sections 2.7, 2.8).

### 2.4 MRI sessions

During the MRI sessions, the participant will first complete the state-based questionnaire on an iPad (identical to lab session) before completing an MRI safety screening form. Prior to being placed in the scanner, the researcher will review the instructions for the Visceral Interoceptive Attention (VIA) task with the participant. The participant will then complete the MRI scan, which is comprised of a structural scan (MPRAGE), three runs of the VIA task, and a resting state functional scan. Details of the task and scanning parameters can be found below (in sections 2.8.7 and 2.9).

### 2.5 Participants

Participants are two naturally cycling women, with a regular menstrual cycle approximately between 27 and 29 days. Recruitment was conducted using flyers posted around the UCLA campus and associated buildings, email listservs, and online website postings. One participant is a healthy control and the other met a clinical threshold for elevated PMDD symptoms. PMDD symptoms were screened using the premenstrual symptoms screening tool (PSST; Steiner et al., 2003). Neither participant reported any form of hormonal birth control, previous pregnancy, current diagnosis of a psychiatric disorder, nor any current use of psychoactive medications. The recruited participants both live within the greater Los Angeles area, are between the ages of 22-23 years, and identify as non-Hispanic (one participant identifies as White, and the other as Asian).

### 2.6 Questionnaires

#### 2.6.1 Baseline questionnaires

A set of questionnaires will be administered online during the baseline session to better characterize each participant. Details of those questionnaires can be found in Table 1.

**Table 1:** Baseline questionnaire details.

Questionnaire	Construct	# of Items	Scoring	Scale Range	Reference
Interoceptive Accuracy Scale (IAS)	Interoceptive Sensibility	21	Total Score	21-105	Murphy et al. (2020)
Multidimensional Assessment of Interoceptive Awareness (MAIA-2)	Interoceptive Sensibility	37	8-subscales	0-5	Mehling et al. (2018)
Interoceptive Confusion Questionnaire (ICQ)	Interoceptive Sensibility	20	Total Score	20-100	Brewer et al. (2016)
Body Perception Questionnaire -Very Short Form (BPQ-VSF)	Interoceptive Sensibility	12	Total Score	12-60	Porges (1993)
Anxiety Sensitivity Index (ASI-3)	Anxiety Sensitivity	18	3-Factors	0-24	Taylor et al. (2007)
Beck Depression Inventory (BDI-II)	Depressive Symptoms	20	Total Score	0-60	Beck et al. (1996)
Patient Health Questionnaire (PHQ-9)	Depressive Symptoms	9	Total Score	0-27	Kroenke et al. (2001)
Perth Alexithymia Questionnaire (PAQ)	Alexithymia	24	Total Score & 5-subscales	24-168	Preece et al. (2018)
Toronto Alexithymia Scale (TAS-20)	Alexithymia	20	Total Score & 3-subscales	20-100	Bagby et al. (1994)
Premenstrual Symptoms Questionnaire (PSQ)	Premenstrual Symptoms	14	Total Score	14-56	Takeda et al. (2006)
Generalized Anxiety Disorder (GAD-7)	Anxiety Symptoms	7	Total Score	0-21	Spitzer et al. (2006)
Health Questionnaire	Health	27	-	-	-
Five Facet Mindfulness Questionnaire (FFMQ-15)	Mindfulness	15	Total Score & 5-subscales	15-75	Baer et al. (2008)
Questionnaire of Unpredictability in Childhood (QUIC)	Early-life adversity	38	Total Score & 5-subscales	0-38	Glynn et al. (2019)
Childhood Trauma Questionnaire (CTQ)	Early-life adversity	6	Cumulative Score	0-42	Pennebaker and Susman (1988)
Demographics	Demographics	13	-	-	-

**Note.** List of questionnaires participants will complete during the baseline session. All measures will be collected remotely. For each questionnaire, we categorized the construct it assesses, the total number of items, whether the scoring generates total scores and/or subscale scores, the minimum and maximum possible scores, and the original reference. For questionnaires with total scores and subscale scores, the total score ranges are reported. Questionnaires without references were lab generated.

#### 2.6.2 State questionnaires

At the beginning of each session, participants will complete a set of state-based questionnaires (i.e., how they are feeling on the present day). The following constructs will be assessed using continuous visual analog scales (VAS): *valence*, *arousal/activation, fatigue*, and the following questions will also be assessed with a VAS: *How depressed do you feel today?, How anxious do you feel today?, How well can you sense your body today?, How in-touch with your body are you today?, How much are you worrying about your body sensations today?, How stressed do you feel today?* Participants will also report whether they feel sick (yes or no) and the amount and time since they last consumed: *food, caffeine, alcohol, diet pills, sleeping pills,* and *tobacco*.

#### 2.6.3 Menstruation reporting

In addition to the LH tracking, participants will report the start and end dates of menstruation for the time preceding data collection and throughout their enrollment in the study. These self-reported dates will be used to align data to the start of each menstrual cycle (first day of menstruation).

### 2.7 Behavioral tasks

#### 2.7.1 Heartbeat Detection task (HDT)

Participants will complete a Whitehead Heartbeat Detection Task (HDT; Kleckner et al., 2015; Whitehead et al., 1977). At the beginning of the task, participants will complete a 2-min echocardiogram (ECG) baseline. For this study, the task will contain 40 trials total with 10 tones for each trial. For a given trial, all ten tones will be presented “in-sync” with the participants heartbeat (200ms after each R-wave) or “out-of-sync” with the participants heartbeat (500ms after each R-wave). After a set of 10 tones, participants will make a forced-choice judgement of whether the tones were presented at the “same” time as their own heartbeats or at a “different” time than their own heartbeats. Following the same/different decision, participants will then make a rating of their confidence in their response using a VAS from *Not at all sure* to *Extremely sure*. A measure of cardiac interoceptive sensitivity can be calculated using a non-parametric signal detection measure: A’ Normalized (Aaronson and Watts, 1987; Khalsa et al., 2009). This measure is optimized for low-signal tasks and captures signal detection ability by comparing the hit rate (number of “same” responses on in-sync trials) to the false alarm rate (number of “same” responses on out-of-sync trials).

Participants will also complete an exteroceptive version of the HDT. For this task, a flashing fixation cross will be presented on for 500ms and off for 500ms (e.g., similar timing of systole and diastole of a 60 beats-per-minute heartrate). An auditory tone will then be played either 200ms (in-sync) or 500ms (out-of-sync) after the onset of the fixation cross. Participants will make the same forced-choice decision as the interoceptive version, but for only 10 trials (5 of each trial type; 10 tones per trial). The task will serve primarily as an attention check, but the same signal detection measure as the interoceptive version of the task can be calculated.

#### 2.7.2 Water Load task (WL)

As a measure of gastric interoception, participants will complete a Water Load (WL) Task (Van Dyck et al., 2016). During this task, participants will be provided with a 1 gallon (∼3.79L) opaque jug of room temperature water and instructed that the researcher will leave for the room for 5 minutes and during that time they are to drink until they feel “*satiated* – *the comfortable sensation you perceive when you have eaten a meal and you have eaten enough, but not too much.*” After 5-min, the researcher will return and provide the participant with a new jug of water and instruct the participant that the researcher will again leave the room for 5 minutes and this time the participant should drink until “*your stomach is completely full and feel that you cannot drink anymore*.” After another 5 minutes, both jugs will be weighed to measure the *amount* of water consumed. For participant safety, each jug is filled with only 1.5L of water. A measure of gastric interoception can be calculated by comparing the ratio of amount of water needed to reach satiation to the total amount of water consumed (i.e., satiation mL/(satiation mL + fullness mL)).

#### 2.7.3 Respiratory resistance task (RR)

As a measure of respiratory interoception, participants will complete a respiratory resistance (RR) task (Wilzok et al., 2023). During this task, participants will wear a mask covering their nose and mouth connected to a two-way non-rebreathing valve (Ambu Mark III, Ambu GmbH), which separates inhalation from exhalation. The exhalation valve will be left open, while the inhalation valve is attached to plastic tubing. At the other end of the tube, the researcher will place different filters (Hans Rudolph Linear Resistors 7100R) that increase the difficulty to inhale. For this study, there will be 5 different levels of difficulty (0 (no filter), 5, 10, 35, and 50 cmH_2_0/L/s). Participants will be exposed to each difficulty for 10 seconds and the presentation order of difficulty will be fully randomized for each session. After each 10 second trial, participants will make VAS ratings of: *subjective effort, unpleasantness, intensity, difficulty, stress,* and *fear*.

#### 2.7.4 Breath hold task (BH)

As a measure of affective response to respiratory challenge, participants will complete a maximum duration inspiratory breath hold (Asmundson and Stein, 1994; Lapidus et al., 2020). During this task they will be instructed to place a clip over their nose and then to “*exhale all air out of your lungs, then take a deep breath in and then hold it as long as you can tolerate*.” The trials will last a maximum of 2 minutes, and the participant will exhale into a capnometer. After the breath hold, the participant will complete a set of VAS ratings on: *subjective effort, unpleasantness, intensity, difficulty, stress, breathlessness, urge to breathe, feelings of suffocation,* and *suffocation fear*. Following a 2-min break, the participant will then complete the same procedure for a second time. Each breath hold session will consist of two breath holds total. The primary outcomes for this task will be the VAS ratings, but duration of each breath hold will also be recorded.

#### 2.7.5 Von Frey Filaments task (VFF)

As a measure of somatosensory sensitivity, participants will complete a Von Frey Filaments task (VFF; Ciaramella et al., 2023). During the task participants will be exposed to five levels of somatosensory stimulation to the palm of their dominant hand, with their eyes closed to avoid visual influence of the color or size of the filaments. Von Frey filaments are monofilaments that allow for a fixed and constant level of pressure. The five levels of force (0.07g, 0.4g, 2g, 4g, 300g) will be presented in random order for 10s each. Immediately after each 10s exposure, the participant will rate the intensity of sensation on a continuous VAS that ranges from *Not at all* to *Extremely*.

#### 2.7.6 Taste Test (TT)

For the Taste test, participants will be provided with 5 flavors of jellybeans (*Juicy Pear, Bubble Gum, Buttered Popcorn, Sizzling Cinnamon,* and *Sunkist Tangerine*), one at a time. After eating each jellybean, participants will rate the flavor on the five basic tastes, using a continuous VAS for each basic taste (sweet, sour, salty, bitter, and umami). In between each jellybean, participants will rinse their mouth with water, and the presentation order of flavors will be fully randomized for each session.

#### 2.7.7 Visceral Interoceptive Attention task (VIA)

During the neuroimaging sessions, participants will complete a Visceral Interoceptive Attention (VIA) task while in the scanner (Adamic et al., 2024; Simmons et al., 2013). Participants will complete 3 runs of the VIA task in the MRI machine during each MRI session. During each run, participants will be presented with written cues to different body locations (“Heart”, “Lungs”, “Uterus”, “Target” (exteroceptive condition)). They will be instructed to “*focus your attention on any sensations you may feel in that part of your body*” and “*to continuously evaluate the sensations from that part of the body*” while “*not attempting to modulate*” that part of their body with their eyes open during the 8s trials (see Table 2 for breakdown of task timing). Additionally, participants will complete a simple attention check (intensity ratings) following 1/3 of trials to make sure they are still engaging in the task. Stimuli will be presented in a fixed-random order across sessions, with each run having a different order (i.e., 3 versions of the task, each presented once per session).

**Table 2:** Via task timing.

Condition	Duration (s)	Repetitions (n)	Total Time (per Run; s)	Overall Time (x3 Runs)
Heart Trial	8s	6	48s	144s
Lung Trial	8s	6	48s	144s
Uterus Trial	8s	6	48s	144s
Exteroceptive	8s	6	48s	144s
Attention Check (Intensity)	4s	8	32s	96s
ITI	Mean 4s (Range: 2-6s)	24	96s	288s
**TOTAL (s)**			324s	972s

**Note.** Exact timing of each component of the Visceral Interoceptive Attention (VIA) Task. Participants will view either a heart, lung, uterus, or exteroceptive trial for 8s, followed by an attention check (1/3 of trials; n = 8 per run; 4s to complete) and then a 4s ITI (fixation cross). Each run will have 24 trials (6 of each condition). Participants will complete 3 runs per session. All times are reported in seconds (s).

### 2.8 Neuroimaging

During the neuroimaging sessions, participants will complete a localizer scan, a MPRAGE, three runs of the VIA task, and a resting state fMRI (rs-fMRI) scan for a total scanning time of 29:57 min. Scans will always be collected in the same order. All scans will be collected using a Siemens 3 T PRISMA with a 32-channel head coil.

#### 2.8.1 Structural scans

The following parameters will be used for the MPRAGE scan: 1900ms repetition time (TR), 2.48 echo time (TE), 9° flip angle (FA), 256 mm field of view (FOV), 208 slices (1 mm thickness), 4:33 min acquisition time (TA).

#### 2.8.2 Functional scans

The following parameters will be used for all functional MRI scans: echo-gradient EPI, acquired axially A ≫ P, 2000ms TR, 30ms TE, 75° FA, 3.4 x 3.4 × 4.0 mm^3^ voxel size, 220 FOV, 33 slices (4.0mm thickness), interleaved acquisition. For each run of the VIA task, the scan will have an acquisition time of 5:28 min (162 vol). The rs-fMRI scan will have an acquisition time of 7:00 min (208 vol). A fixation cross will be presented during the rs-fMRI and participants will be instructed to keep their eyes open, but blink normally.

### 2.9 Biological samples

#### 2.9.1 Stool

Following each MRI session, participants will be provided with an Omnigene Gut Kit from DNAGenotek to take home with them. They will complete the stool sample at home and return the stool kit at one of their following sessions. The stool will be processed using shotgun metagenomic sequencing, using Illumina DNA Prep and sequencing with the NovaSeq X Plus platform (2 ×150 bp paired-end reads, ∼20 million paired-end reads per sample, ∼6GB). This approach allows for the characterization of the gut-microbiome at the species and functional potential levels.

### 2.10 Analytical approach

The sample size of this study will preclude many traditional approaches to statistical significance testing. However, we plan to use within-participant modeling to test if there is significant variation across the menstrual cycle in all our dependent variables for each participant, and then descriptively compare the patterns of results between the control and PMDD participant. A list of key measures and their mapping onto constructs and aims can be found in Table 3. Specifically, we plan to use Generative Additive Models (GAMs) to model fluctuations across the menstrual cycle, which is a statistical approach applicable to single-subject, densely-sampled designs (Pritschet et al., 2024). The GAM models will use a cyclic cubic spline, which fits the model such that the first observation and last observation are adjacent to each other. We will also limit the number of splines (approximately 4 splines maximum) to prevent overfitting of the data. We will also control for the cycle number (i.e., month 1-4) which will remove practice effects or slow-moving temporal drifts not associated with the menstrual cycle. Finally, we plan to conduct sensitivity analyses where we will also control for state variables (e.g., sleep, caffeine, stress, alcohol consumption etc.) within the GAMs.

**Table 3:** Primary measures and construct mapping.

Task	Primary Measure	Construct	Organ System	Relevant Aim
Self-Report Questions	Interoceptive Sense	Interoceptive Sensibility	Whole Body	Aims 1, 2, & 3
	Interoceptive Worry	Interoceptive Sensibility	Whole Body	Aims 1, 2, & 3
	Affective Valence	Mood	NA	Aims 2 & 3
	Anxiety	Mood	NA	Aims 2 & 3
	Depression	Mood	NA	Aims 2 & 3
Heartbeat Detection	A’ Normalized	Interoceptive Accuracy	Cardiovascular	Aims 1, 2, & 3
	Bias (c)	Interoceptive Bias	Cardiovascular	Aim 1
	Confidence	Interoceptive Meta-awareness	Cardiovascular	Aim 1
Water Load	Water Consumption Ratio	Interoceptive Accuracy	Gastric	Aims 1, 2, & 3
Respiratory Resistance	Difficulty Ratings	Interoceptive Accuracy	Respiratory	Aims 1, 2, & 3
Breath Hold	Difficulty/Fear Ratings	Interoceptive Appraisal	Respiratory	Aims 1, 2, & 3
Von Frey Filaments	Intensity Ratings	Exteroceptive Sensitivity	Somatosensory	Aims 1, 2, & 3
Taste Test	Flavor Ratings	Exteroceptive Sensitivity	Gustatory	Aim 1
Visceral Interoceptive Attention	Functional Contrasts	Interoceptive Neural Representation	Neural	Aim 1
Structural MRI	Cortical Thickness	Interoceptive Brain Structure	Neural	Aim 1
Resting-State fMRI	Functional Connectivity	Interoceptive Brain Function	Neural	Aim 1
Stool Sample	Alpha Diversity & Differential Abundance	Gut-Microbiome Characteristics	Gut-Microbiome	Exploratory Aim

**Note.** The above listed metrics are not exhaustive but represent many of the key metrics that can be calculated from the measures, the construct they map onto, and the aims which they will be relevant to.

The structural MRI (sMRI) data will be processed using the Freesurfer longitudinal pipeline (Dale et al., 1999; Fischl, 2012). This pipeline is optimized for repeated sMRI images from a single participant. We will then extract cortical thickness values using an ROI approach and use the cortical thickness of each ROI at each timepoint as the outcome variable of the GAM described above. The fMRI data will be preprocessed using fMRIPrep (Esteban et al., 2019). The resting state scans will be used to test if there are fluctuations in resting state functional connectivity across the menstrual cycle. Data from the VIA task will be analyzed by contrasting the interoceptive conditions against the exteroceptive condition to examine changes in interoception related activation across the menstrual cycle. We will also contrast the interoceptive conditions against each other to probe changes in organ-specific interoception-related activation across the menstrual cycle.

## 3 Discussion

The CHIRP study will be the first step in addressing a fundamental gap in reproductive and interoceptive neuroscience—whether cyclical hormonal transitions reorganize the sensing and interpretation of bodily signals and their coupling to affect. By combining dense within-person sampling with multimodal assessments across cardiovascular, respiratory, and gastrointestinal systems, this study is designed to disentangle short-term measurement variability from phase-linked modulation of interoceptive processing. Such deep phenotyping is necessary to characterize whether interoception represents a plausible pathway through which the menstrual cycle modulates mood symptoms.

A central contribution of this protocol lies in its measurement framework. Interoception is multidimensional and organ-specific, yet most prior work has relied on single-method, single-system approaches. The CHIRP battery is the first to integrate self-report, task-based accuracy, and insight measures across multiple organs, allowing evaluation of cross-system concordance and reliability over time. This data will inform which interoceptive indices are sufficiently stable (or dynamic) to serve as targets in future mechanistic and clinical studies. Such a battery can serve as a more general template for deeply characterizing profiles of interoceptive functioning in other studies of interoception. Furthermore, the dense-sampling design also enables questions to be asked that are not tractable in traditional cohort studies. Repeated assessments across four months permit estimation of intra-individual effect sizes, temporal coupling between interoception and mood, and potential lagged relationships surrounding hormonally sensitive windows such as the late-luteal transition.

The strengths of this study should be considered in the context of its limitations. While the sample size (N = 2) will preclude population-level generalization, it is well suited for hypothesis generation and for identifying personalized patterns of interoceptive change, an approach that has proven valuable in prior deeply phenotyped studies (Poldrack et al., 2015; Pritschet et al., 2020, 2024). Additionally, the participants will be asked to report on the start and end dates of menstruation but will not be asked to provide information on flow characteristics (such as heaviness of flow and pain), which is a potential limitation. To reduce participant burden, the standardized questionnaires will only be administered during the baseline session, and single item VAS questions will be used to assess self-reported mood and interoception throughout the study. While these single item VAS questions have not been validated in the same way as the full length standardized measures, we are primarily interested in within-person changes, which these items should be well-suited to capture.

Overall, the CHIRP study will aim to improve our understanding of interoception generally and within the context of the menstrual cycle. From a translational perspective, this protocol may provide indications about whether altered body sensing represents a modifiable mechanism in PMS/PMDD. Identifying when—and in which organ systems—interoceptive signals most strongly relate to affect could guide the timing of assessments, selection of outcome measures, and development of targeted interventions aimed at recalibrating interoceptive inference. In summary, the CHIRP study will establish a platform for interrogating the dynamic interface between reproductive hormones, interoception, and mood. The resulting dataset is intended to refine theory, improve measurement strategies, and inform the design of larger confirmatory studies focused on menstrual-cycle–linked mental health.

## CRediT authorship contribution statement

**Paul W. Savoca:** Writing – original draft, Visualization, Project administration, Methodology, Investigation, Formal analysis, Data curation, Conceptualization. **Savannah D. Lopez:** Writing – review & editing, Investigation. **Sahib S. Khalsa:** Writing – review & editing, Supervision, Methodology, Conceptualization. **Bridget L. Callaghan:** Writing – review & editing, Supervision, Methodology, Conceptualization.

## Ethics declaration

Written informed consent to take part in the study and to publish the article has been obtained from all participants or their legal representatives. The privacy rights of participants have been observed.

This study included organ or tissue donors. This study includes human biological material and consent was obtained by donors, or their next of kin or legal representatives, for use in this study and for publication of the article. The samples used in this research were not sourced from executed prisoners or prisoners of conscience.

This study was performed in compliance with relevant laws, regulatory frameworks and guidelines where the research took place. This study was approved by the University of California, Los Angeles (UCLA) IRB. (Approval No. IRB-24-6193)

## Funding

Paul W. Savoca was supported by the National Science Foundation Graduate Research Fellowship Program under Grant No. DGE-2034835. Any opinions, findings, and conclusions or recommendations expressed in this material are those of the authors and do not necessarily reflect the views of the National Science Foundation.

## Declaration of competing interest

The authors declare that they have no known competing financial interests or personal relationships that could have appeared to influence the work reported in this paper.

## Data Availability

Data will be made available on request.
